# Transactions of the Chicago Gynæcological Society

**Published:** 1885-11

**Authors:** 


TRANSACTIONS OF THE CHICAGO GYNÆCOLOGICAL SOCIETY.

*Annual Meeting, Friday evening, 16th October, 1885.* The PRESIDENT, DR. H. P. MERRIMAN in the chair.

The following reports were read:

I. REPORT OF THE SECRETARY.

TO THE FELLOWS OF THE CHICAGO GYNÆCOLOGICAL SOCIETY.

*Gentlemen:* Summary of work of the SOCIETY for the sixth year, ending September 22nd, 1885.

There have been eleven regular meetings held, at which the average attendance has been eleven.

The following communications have been made to the Society:

1. Address by the RETIRING PRESIDENT, DR. A. REEVES JACKSON, on THE REQUIREMENTS AND GENERAL OUTLOOK OF THE SOCIETY.

2. THE GENERAL SUBJECT OF ABORTION, by Dr. Edward Warren Sawyer.

3. TWO CASES OF INTERSTITIAL PREGNANCY, with exhibition of the specimens, by Dr. W. H. Byford.

4. REPORT OF AN INTERESTING CASE OF DOUBLE OVARIOTOMY, by Dr. E. C. Dudley.

5. DR. CHRISTIAN FENGER'S REPORT ON DR. W. H. BYFORD'S TWO CASES OF MURAL PREGNANCY.

6. INTRA-MURAL LEIO-MYOMA, specimen presented by Dr. E. C. Dudley.

7. THE FUNCTIONS OF THE MEMBRANES IN LABOUR, by Dr. Henry T. Byford.

8. THE INFLUENCE OF CIMICIFUGA RACEMOSA UPON PARTURITION, by Dr. J. Suydam Knox.

9. SOME REMARKS ON THE VALUE OF PERMANGANATE OF POTASH IN AMENORRHŒA, by Dr. Edward J. Doering.

10. CHRONIC PERI-UTERINE ABSCESS, by Dr. C. Fenger.

11. REMARKS UPON A TERATOM, with exhibition of specimen, by Dr. W. W. Jaggard.

12. TWO UNUSUAL CASES IN OBSTETRICS, by Dr. Charles Caldwell.

13. THE NORMAL POSITION OF THE UTERUS AND ITS RELATION TO THE OTHER PELVIC ORGANS, by Dr. Franklin H. Martin.

14. A REPORT OF A CASE OF A FŒTUS, ENCLOSED IN ITS SISTER'S PLACENTA, by Dr. James H. Etheridge.

15. EXHIBITION OF A PLACENTA WITH CALCAREOUS DEPOSITS, by Dr. James H. Etheridge.

16. RPORT OF A CASE OF LEIO-MYOMA OF THE VAGINA AND UTERUS, by Dr. Henry T. Byford.

17. SHOCK AND NERVOUS INFLUENCE IN PARTURITION, by Dr. H. P. Newman.

18. DIET AND MANAGEMENT OF ARTIFICIALLY FED INFANTS, by Dr. A. H. Foster.

19. ABDOMINAL AND GYNÆCOLOGICAL SURGERY IN ENGLAND, SCOTLAND AND HEIDELBERG, by Dr. E. C. Dudley.

At the July meeting the constitution was duly amended, changing the limit of membership from 20 to 25.

Upon ballot, the name of one Fellow was removed from the roll.

Six new Fellows have been elected.

Total membership, 22.

At the meeting in March, it was voted by the Society to issue a certificate of membership to each Fellow of the Society, which has been duly executed.

The Secretary cannot terminate this, his fifth annual report to this honorable body, without congratulating the Fellows upon the advancement made; surely no other year of its existence has been marked by a greater degree of advancement. This he believes no less due to the constant and efficient service of the President than to the excellent and faithful reports of the Society's Editor.

Respectfully submitted,

EDWARD WARREN SAWYER, M. D.,

*Secretary.*

II. REPORT OF THE EDITOR.

TO THE FELLOWS OF THE CHICAGO GYNECOLOGICAL SOCIETY.

*Gentlemeu:* The editor begs permission to submit the following report for the year ending Friday evening, 16th Oct., 1885.

Copies of the proceedings of each meeting of the CHICAGO GYNECOLOGICAL SOCIETY, with the exception of that of 19th April, have been, or will be, sent to the following medical journals:

1. PHILADELPHIA:
   *The Medical News.*
2. NEW YORK:
   *The American Journal of Obstetrics,*
   *The New York Medical Journal.*
3. BOSTON:
   *The Boston Medical and Surgical Journal.*
4. BALTIMORE:
   *Maryland Medical Journal.*
5. CINCINNATI:
   *The Obstetric Gazette.*
6. LOUISVILLE:
   *The American Practitioner.*
7. CHICAGO:
   *The Journal of the American Medical Association,*
   *The Chicago Medical Journal and Examiner.*
8. ST. LOUIS:
   *The Weekly Medical Review.*
9. NEW ORLEANS:
   *New Orleans Medical and Surgical Journal.*
10. SAN FRANCISCO:
    *Pacific Medical and Surgical Journal.*
11. LONDON:
    *The British Gynæcological Journal.*

The editor desires to suggest the propriety of including a German and a French journal in the list.

The report of the proceedings of the meeting of the 19th April appeared only in the CHICAGO MEDICAL JOURNAL AND EXAMINER, the official organ of the SOCIETY, for the reason that DR. FENGER subsequently presented the same subject,—Chronic Peri-uterine Abscess,—before the SECTION on OBSTETRICS, AMERICAN MEDICAL ASSOCIATION, at the annual meeting in New Orleans.

During the year, two formal complaints, with reference to the accuracy of the reports of the transactions, have come to the editor's knowledge.

The first complaint,—a serious misrepresentation,—was sent to the editor in conformity with the dictates of courtesy and parliamentary usage. The correction was at once transmitted to the editor of the *American Journal of Obstetrics* and appeared in the September number of that periodical.

The second complaint,—an alleged misrepresentation,—was sent directly to the editor of the *American Journal of Obstetrics.* As the well-known rule, with reference to the transmission of corrections was disregarded in this case, the complainant's communication was referred by DR. MUNDÉ to the SOCIETY'S editor. Correspondence ensued. The SOCIETY'S editor stated that he was under the conviction that the complainant actually uttered the words printed in the the report of the transactions. DR. MUNDÉ accordingly refused to print the alleged correction.

The editor desires to call attention to the subject for the reason that such complaints seriously affect the value of the reports of the transactions in the eyes of medical journalists, and will limit their circulation.

The relation of the reporter to the debater in any society is a purely impersonal one. Inobservance of the conventions, naturally suggested by courtesy and good breeding, upon the part of any member towards the reporter, ought never to be regarded as a personal matter. Nor does the editor, in the present instance, construe any unpleasant occurrence during the year in the light of a personal offence.

It is certainly a very trying matter to see one's remarks emasculated, distorted beyond all recognition, utterly senseless, widely circulated in the medical journals. When such misrepresentation is due to the carelessness, stupidity or perversity of the scribe, he deserves to be censured. As a matter of fact, he usually comes in for a full share of vituperation, whether merited or not. But the fault is not always the reporter's. The debater, himself, may sometimes be in error.

In all discussions there is much desultory and irrelevant talk. Debaters are usually prodigal in the use of words. Only masters of the art "skim over a multitude of circumstances under which the occurrence has taken place, because they are aware that it is proper to reject what is only accessory to the object which they would present in prominence." A peculiar involuntary association of ideas is not infrequently observed. Men "are" usually "held in thraldom to the order and circumstances in which their perceptions were originally obtained." The great observer of human nature has not failed to notice this fact. "Mrs. Quickly, in reminding Falstaff of his promise of marriage supplies a good example of this peculiarity." "Thou didst swear to me upon a parcel-gilt goblet, sitting in my Dolphin chamber, at the round table, by a sea-coal fire, upon Wednesday in Whitsun week, when the prince broke thy head for likening his father to a singing man of Windsor,"—and so forth. (Sir William Hamilton.)

Medical discussions, in particular, are marred by verbosity and wandering from the subject. The debates in the *symposia* of the CHICAGO GYNÆCOLOGICAL SOCIETY, during the past year, have not been entirely free from these faults.

Remarks, made in the heat of discussion, are not always characterized by accuracy of statement. A short time since, an American gynæcologist, — as universally beloved for personal qualities as he is famed for professional skill, — made the remarkable assertion that he had observed several cases ot epithelioma of the *glans penis* result from copulation with women whose cervices were the seat of carcinoma. A mortifying retraction of the statament followed, and the heat of discussion was urged in palliation.

Ideas are not always expressed *clearly*, *distinctly*, and *adequately*. Notions are frequently *obscure* and *confused*. That celebrated mathematician and philosopher, LEIBNITZ, published a small treatise in 1684 on the *necessary characters* of PERFECT KNOWLEDGE. MR. BATNES has appended to his translation of the *Port Royal Logic* an excellent translation of LEIBNITZ's tract. A study of this translation, by the FELLOWS of the SOCIETY, would materially ease the reporter's shoulders of his load. Then there exists in the minds of many medical gentlemen a supreme contempt for the science of the formal or necessary laws of thought. It is the inalienable right of every American citizen to think and talk as it pleases him! America is a big, rich, free, powerful country. The American medical mind evinces a natural fondness for the universal proposition, affirmative or negative. Major premise, minor premise, and conclusion, are frequently combined in utter defiance of the laws of the syllogism. Fallacies, — logical and material, — exceed, in number and degree, those described by such systematic writers as SIR WILLIAM HAMILTON and JOHN STUART MILL.

The "accidence and syntax" of English grammer is not always observed, and DR. CAMPBELL's canons are sometimes held in derision.

It is by no means an easy task to construct out of such an incongruous mass of materials a clear, distinct, concise report, which will reflect credit upon the participants in the debate, and, at the same time, truthfully represent the discussion.

In order to secure the fairest possible representation of each debater, the following plan, with reference to the reports of the transactions, has been pursued in the last two meetings. The FELLOWS were requested to send *résumés* of their remarks to the editor, within one week after the meeting.

These condensed statements have been printed as written by the speakers themselves.

The remarks of the gentlemen who failed to comply with this request have been omitted.

This plan is manifestly imperfect, and will not furnish an accurate record of the proceedings. Memory is proverbially treacherous. At home, in the library, away from the heat of discussion, statements will be modified, retracted, and the whole tenor of the discourse radically altered. The plan, however, is designed to conciliate the FELLOWS, and thus promote the welfare of the SOCIETY.

The editor desires to suggest the propriety of the appointment of a Committee on Publication, in addition to the reporter. The reports, revised and signed by the editor and Committee on Publication, will thus constitute official documents, beyond which there is no appeal.

In conclusion, the editor desires to gratefully acknowledge the charity with which, in general, the FELLOWS of the SOCIETY have judged his reports.

Respectfully submitted,

W. W. JAGGARD, M. D.,

Editor.

III. THE ADDRESS OF THE PRESIDENT, DR. H. P. MERRIMAN.

TO THE FELLOWS OF THE CHICAGO GYNÆCOLOGICAL SOCIETY.

*Gentlemen:* At this our annual meeting, it seems appropriate to cast our eyes backward upon the meetings of the year past, to see how faithful we have been and what we have accomplished. Such a review is always profitable, as it tells us where we stand, and what we may expect the future to bring to us.

I think myself peculiarly fortunate in having had for my predecessors in the presidency far-seeing, thoughtful men, who have built so wisely that our SOCIETY grows stronger and more vigorous from year to year. You all remember the wise words uttered a year ago by the retiring president, advising the course which the Society has pursued this year, and which has proved so advantageous. During the past year no meeting has failed. Even in that worst of snow-storms, when the street cars did not run, we had a well-attended and instructive meeting at the residence of DR. DUDLEY. The interest of the members has been shown not only in their attendance, but there has been no lack in the papers read. Every evening we have had one and sometimes two papers, with a profitable discussion upon each; and a constantly increasing interest has been shown by the Fellows in preparing themselves for the discussions, by studying up the subjects announced for each evening. The vote that the meetings should be continued late into the summer also shows the interest of our Fellows. We have frequently had invited guests, who have expressed a thorough appreciation of the earnest work of our Society.

I feel, gentlemen, that we can congratulate ourselves upon a successful year; and it is but fair that the credit should be awarded where it belongs. All have worked well, but to our secretary is due a large share of the credit for the past year's success. He has been indefatigable in getting contributions of interest from the Fellows, to say nothing of his promptness in sending notices to the members of our meetings, and of his assistance to each entertainer in arranging those little matters of refreshment which add so much to our intellectual enjoyment. Another and most effective agency has been the excellent reports our editor has made of the work of the Society. They have been promptly printed, not only in our own city, but also in the leading cities of our country, and lately even in London and Paris. I feel that to Dr. Jaggard, more, perhaps, than to any other one person, is due the success of the Society this year. These two offices,—of Secretary and Editor,—are of the greatest usefulness to the Society. It is important that they be well filled, and I do not see how we can improve the present occupancy.

I do not desire to make a report upon the progress of gynæcology, and there is not time if I did wish it; but I cannot avoid a feeling of amazement and admiration at the rapid growth of knowledge in this departmeut of medicine. One subject after another has been taken up and investigated: every year adding greatly to our knowledge of the nature of the disorders and the methods of relieving them. Surgery, especially, has made gigantic strides. New methods and greater skill have reduced the mortality in the capital operations to such a minimum, particularly in ovariotomy, that the belief is rapidly gaining foothold that abdominal surgery, even in the male, should be performed by the ovariotomist. The operations upon the perineum, the vagina, the cervix and the vesico-vaginal septum are now nearly perfect. Gynæcology owes almost everything to surgery, and surgeons are to-day doing the best work done in elucidating the pathology and treatment of diseases of the pelvic organs. There is, however, danger of our thinking that only a surgeon can do anything for the relief of woman's diseases. All honor to surgeons. It seems to me, however, that there is a large field for investigation and profit in diseases of women that are not surgical; that all the sufferings of women are not due to textural changes in their reproductive organs. These parts are so abundantly supplied with nerves and vessels,—are so closely connected with both the sympathetic and spinal nervous systems, that they act as excitants and recipients of suffering. No organs in the body are more subject to sympathetic or reflex influence, or feel more keenly any influence brought to bear upon them. Fatigue, exposure, debility, depressing emotions, disorded blood states, constitutional cáchexias, or the various diatheses are sure to have their influence on these important organs in the way of producing suffering which cannot be relieved by surgery. These subjects ought to receive more attention. The neuroses of the pelvis have not been sufficiently studied. Uterine and ovarian neuralgia are still vague terms. We do not know when dysmenorrhœa is due, and when not due, to textural change in the ovaries. For this painful complaint women have been deprived of these organs and no disease could be found in them. This is like extracting sound teeth for *tic-douloureux*. Cannot our Fellows the coming year bring forward some additional light upon these obscure neuroses? Then, how little we know of sterility and its cure. We see certain surgical defects that may be remedied, but there is room for immense improvement in this subject. The subject of functional diseases, too, is not half understood. Turn where we will, opportunities offer for earnest work, and I trust they will be improved the coming year more than at any time in the past.

As I see the work we *have* done, I realize what we *may* do, and I desire to have our SOCIETY take such a stand and have such an influence that *one* of the proudest titles any one of us can append to his name will be "Fellow of the Chicago Gynæcological Society."

### IV. ELECTION OF OFFICERS.

The following officers were elected for the year 1885–6.

PRESIDENT, Dr. Daniel T. Nelson.

FIRST VICE-PRESIDENT, Dr. Henry T. Byford.

SECOND VICE-PRESIDENT, Dr. Charles Warrington Earle.

SECRETARY and TREASURER, Dr. Edward Warren Sawyer.

EDITOR, Dr. W. W. Jaggard,

### V. BANQUET.

The Fellows were then entertained at an elaborate banquet in the Calumet Club, by the retiring PRESIDENT, DR. H. P. MERRIMAN.

W. W. JAGGARD, M. D.,

*Editor.*

2330 Indiana Ave., 27th October, 1885.

